# Stress, non-restorative sleep, and physical inactivity as risk factors for chronic pain in young adults: A cohort study

**DOI:** 10.1371/journal.pone.0262601

**Published:** 2022-01-21

**Authors:** Maja Lindell, Anna Grimby-Ekman

**Affiliations:** 1 Chronic Pain, School of Public Health and Community Medicine, Institute of Medicine, Sahlgrenska Academy, Gothenburg University, Gothenburg, Sweden; 2 Biostatistics, School of Public Health and Community Medicine, Institute of Medicine, Sahlgrenska Academy, Gothenburg University, Gothenburg, Sweden; University of Minho, PORTUGAL

## Abstract

**Background:**

Chronic pain is a common condition which causes patients much suffering and is very costly to society. Factors known to be associated with chronic pain include female gender, acute pain, depression, and anxiety. This study investigated whether stress, sleep disturbance, and physical inactivity were risk factors for developing chronic pain among young adults, and whether there were any interactions between these.

**Methods:**

This retrospective longitudinal study was based on an existing database from a cohort study on IT use and health, called Health 24 Years. A questionnaire was sent to students aged 19–24 in Sweden for five consecutive years, containing questions on pain, stress, sleep, physical activity, technology use, health, and more. In logistic regressions, stress, sleep, and physical activity at baseline were potential predictors of chronic pain one and four years later. In addition, a new variable including all possible interactions between potential predictors was created to test for effect modification between risk factors.

**Results:**

At the one-year follow-up, stress, non-restorative sleep, and physical inactivity showed odds ratios of 1.6 (95% CI: 1.0–2.4), 1.5 (95% CI: 1.0–2.3), and 1.8 (95% CI: 1.1–3.0) respectively after adjusting for confounders, the reference being non-stressed, having restorative sleep and being active. At the four-year follow-up, stress showed an adjusted odds ratio of 1.9 (95% CI: 1.3–2.9), while non-restorative sleep and physical inactivity were statistically insignificant. At the one-year follow-up, the interaction between risk factors were significant. The most clear example of this effect modification was to be inactive and not have -restorative sleep, compared to individuals who were active and had restorative sleep, showing an adjusted odds ratio of 6.9 (95% CI: 2.5–19.2) for developing chronic pain one year after baseline. This in comparison of odds ratios for only inactive respectively only non-restorative sleep being 1.7 (95% CI: 0.6–5.3) respectively 1.6 (95% CI: 0.7–3.5).

**Conclusions:**

Stress, non-restorative sleep, and physical inactivity were risk factors for developing chronic pain one year after baseline, and stress were also a risk factor four years after baseline. These findings suggest that non-restorative sleep and inactivity are risk factors in the short term while stress is a risk factor in both the short and the long term. In addition to the independent effects of non-restorative sleep and inactivity, their combination seems to further increase the odds of chronic pain.

## Introduction

Chronic pain is a common public health problem and has a prevalence of approximately 20% in general populations [1]. It has a severe impact on individuals’ quality of life and productivity [2–4], and is also a burden for society in terms of health care costs and inability to work [5]. The International Association for the Study of Pain (IASP) defines chronic pain as “pain that lasts or recurs for longer than 3 months”, and hence differs from acute pain [6]. Chronic pain is likely both developed from and maintained by a combination of neurobiological, psychological, and social factors, and is associated with older age, female gender, socio-economic background, smoking, high comorbidity, mental health issues, obesity, sleep disorders, genetics, and adverse life events in early life [7, 8]. This complex process of developing and maintaining chronic pain is the reason why we want to focus on the factors of stress, sleep and physical activity and how they may interact.

It is widely believed that psychosocial distress, earlier traumas or abuse, depression or sleep problems matter in the development of chronic pain [9, 10], and that perceived stress may have an important role. Individuals with perceived high stress have a higher risk of developing chronic pain in the neck and shoulders as well as persistent back pain, specifically investigated as stress at work [11–16]. Also among younger age groups perceived stress is known to be associated with neck pain and headaches [17]. Acute and chronic emotional distress is associated with the development of fibromyalgia [11], and early life stressors, such as child abuse, have an impact on the probability of developing chronic pain syndromes [9, 18–20]. In the present study stress is defined as perceived stress to capture not the stressors, but rather the experience of stress, incorporated in the psychological stress cycle [21].

The association between chronic pain and sleep disturbance has been well known for a long time [22, 23]. Studies point to a bidirectional occurrence of sleep disturbance and chronic pain, with sleep disturbance preceding the onset and worsening of pain, as well as sleep disturbance resulting from pain [24]. It is likely that sleep disturbance predicts chronic pain to a higher extent than pain predicts sleep disturbance. Sleep disturbance has been shown to precede chronic headaches, fibromyalgia syndrome, and musculoskeletal pain, both widespread and regional [9, 25–29]. It also predicts the probability of pain improvement in chronic pain patients and pain levels on the next day. Pain intensity does not seem to influence sleep disturbance in the far future [26]. One night of complete sleep deprivation has been shown to induce hyperalgesia in healthy subjects, as well as increased anxiety. Sleep quality affects the likelihood of the resolution of chronic widespread pain [30]. In chronic pain patients, sleep and pain can turn into a vicious cycle; sleep deprivation causes increased pain which in turn causes sleep deprivation [31].

Physical activity has previously been shown to be an important health factor in numerous chronic diseases [32]. It is also used as treatment for a number of conditions, such as depression and some chronic pain syndromes [33]. Physical function and pain intensity in chronic pain patients have been shown to improve when treated with physical activity, although the quality of the evidence is relatively low. Exercise is considered a safe treatment option, with few adverse events reported [34]. There are few studies on physical activity as a protective factor for developing chronic pain. One study in mice found that rats with running wheels in their home cages were less likely to develop chronic pain conditions [35]. Some studies on physical activity and lower back/neck/shoulder pain have found that physical activity may be protective [36, 37], while others have shown inconsistent results regarding how leisure-time physical activity affects the risk of lower back pain [38, 39]. One study found that a higher level of physical activity could increase the function of the endogenous pain inhibitory systems [40] which are involved in the pathogenesis of chronic pain syndromes. Physical activity is used to treat different pain syndromes, but has not been thoroughly studied as a protective factor in the development of chronic pain. More research on the subject is needed. Physical activity could be seen as a health factor both for preventing and decreasing pain symptoms. Physical inactivity is a risk factor for health and also a possible risk factor for chronic pain. In younger groups physical inactivity is increasing and hence important to understand as a possible risk factor for chronic pain development.

To our knowledge, prior longitudinal studies investigating the development of chronic pain included persons with pain at baseline, and only excluded those with chronic pain. In this study we specifically focus on chronic pain in young adults. This is of importance due to the fact that stress, sleep disturbance and physical inactivity among young adults are increasing [41–43].

The aim of this study was to investigate whether stress, sleep disturbance, and physical inactivity were risk factors of chronic pain among young adults. Specifically, to see if these risk factors modified the effect of each other.

## Materials and methods

### Cohort data

The data were drawn from a retrospective longitudinal study, the Health 24 Years study, with data collected during 2002–2008, with two recruitment years, one in 2002 and one in 2004. The Health 24 Years study was approved by the local ethics committee at Gothenburg University (ref: Ö-491-01) and followed the Helsinki declaration. All participants were informed of the nature of the study and gave written consent to participate.

In 2002 participants were recruited from the enrollment lists of medical programs and computer science programs of different universities in Sweden. Those attending medical school were from the University of Gothenburg, Lund University, and Linköping University, while those attending computer science programs were from the University of Gothenburg, Chalmers University of Technology, the University of Borås, and the University of Skövde. In 2004, the recruitment also included other programs such as nursing and civil engineering. Students were invited by letter, and two tickets to the cinema were offered as compensation. Reminders were sent to those who did not answer. The invitation and information letter was sent to 1728 people in 2002 and 1697 in 2004; 2471 of these 3425 individuals responded, giving a response rate of 72%.

### Study sample

**Fig 1:**
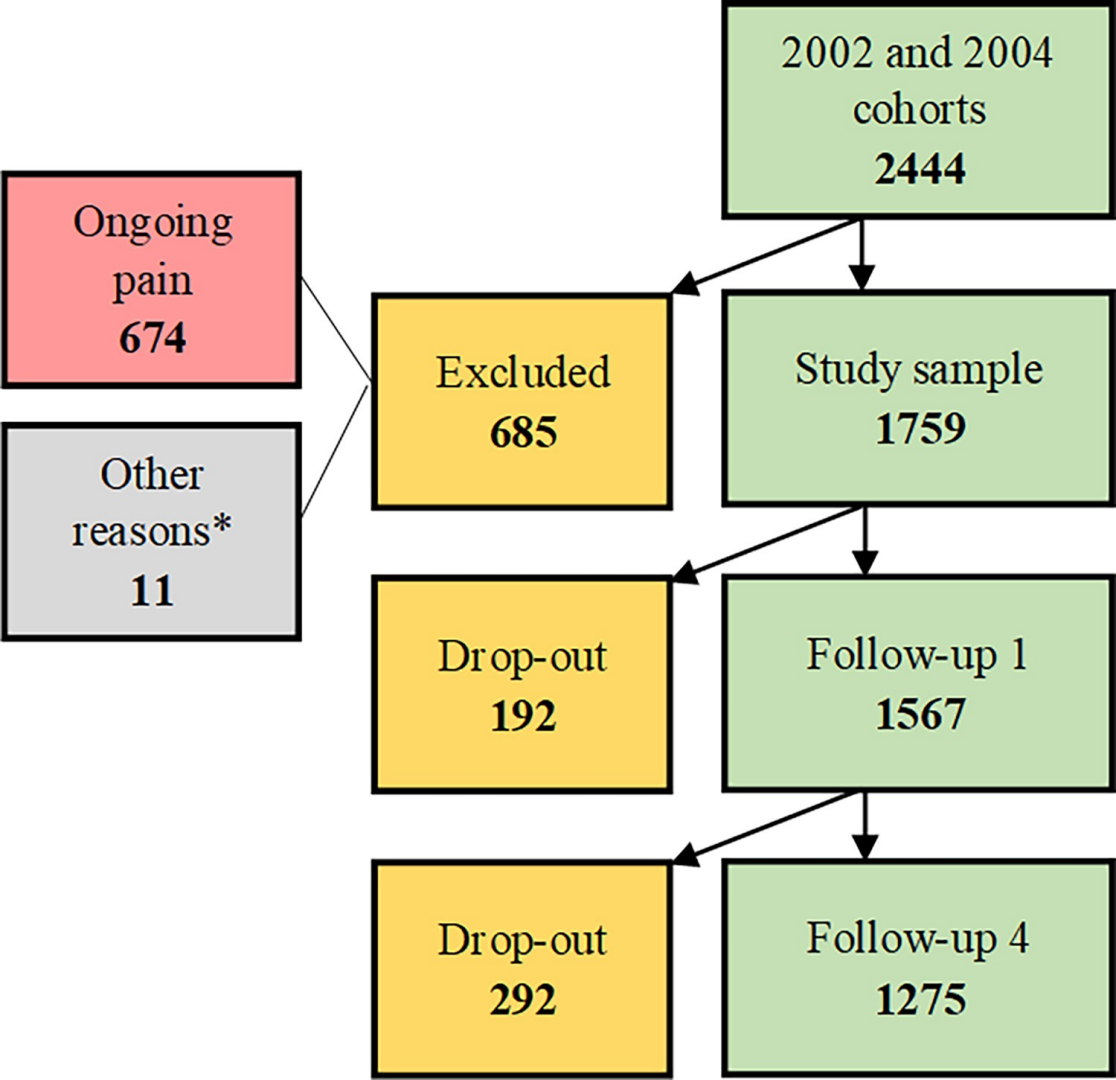
Flowchart of number of responders at different stages in the study. *Individuals excluded due to other reasons than having ongoing pain, e.g. too old, too young, not a university student.

Criteria for inclusion in this study were to be university students, age 19–25 and not having pain at baseline. From the 2471 responders in the cohort data, twenty seven individuals were excluded, as they did not fulfill the inclusion criterion of being aged 19–25 years. Hence, from the cohort 2444 individuals were present at baseline. Of these 2444, 11 individuals were excluded as they had been miss-classified as university students, and another 674 were excluded as they fulfilled the exclusion criterion of having pain at baseline, leaving 1759 individuals included in the study at baseline (1029 men and 730 women). We used the data from the one-year and four-year follow-ups and as this study uses already collected data the variables to analyze were chosen among existing questionnaires from the mentioned cohort. At the one-year follow-up 1567 answered the questionnaires and at the four-year follow-up 1275 answered (Fig 1).

### Questionnaire and study variables

The questionnaires from the cohort, on which our present study is based on, contained questions about demographic factors, nicotine and alcohol consumption, use of computers and other technology, physical activity, stress and demands in school, general health, sleep quality and pain in the back, neck, or upper extremities. A more thorough description of the cohort and the included questions in the questionnaire are described elsewhere [44–46].

The original questions chosen for the main variables are given in **Table 1** (translated from Swedish to English). These variables were dichotomized before being analyzed.

**Table 1:** Original questions selected for the main variables.

**Chronic pain**: Are you experiencing any of the following at present? If yes, give the number of days that this period of symptoms has lasted.
Pain/ache from the upper spine/neck
Pain/ache in the lower part of the spine
Pain/ache in the shoulder/arm/wrist/hands
**Stress**: Stress is defined as “a condition where you feel tense, restless or anxious or can’t sleep at night because you keep thinking about problems”.
In the past 12 months, have you experienced such stress for more than 7 consecutive days? [Yes/No]
**Sleep**: Have you had any of the following problems during the past six months?
Difficulty falling asleep
Frequent awakenings
Not feeling rested when you wake up
Feeling tired or sleepy during the day
[Never/A couple of times a year/A couple of times a month/A couple of times a week/Every day]
**Physical activity**: Approximately how much time did you spend on the following activities in the past 7 days?
[…] Physical activity/Exercise [hours/week]

The general definition used for when acute pain becomes chronic is three months, or 90 days [7]. We defined chronic pain by pain in any of the locations above for more than 89 days.

The question about stress [47], validated by Elo et al [48], was dichotomized in its original version.

The sleep questions, based on a sleep and wakefulness form [49], all had answers dichotomized by categorizing “Never”, “A couple of times a year”, and “A couple of times a month” as “No” and “A couple of times a week” and “Every day” as “Yes”. A previous study [27] found that non-restorative sleep was the strongest predictor of chronic widespread pain, and hence this was chosen as the main variable for assessing sleep in the present study. Hence, we used a variable defined on the question “Not feeling rested when you wake up” (“Never” to “a couple of times a month” were marked as “No” and “a couple of times a week” and “every day” were marked as “Yes”). For a sensitivity analysis additional variables was created to further investigate how different sleep-related issues affected chronic pain. Frequent awakenings and difficulty falling asleep were merged into one binary variable called “sleep disturbance”, where “Yes” stood for having problems with at least one of them, and “No” stood for not having a direct sleep disturbance. Non-restorative sleep and daytime tiredness were merged into another binary variable called “tiredness”, just like the previous one. These alternative sleep variables were run in a model which also included stress and physical inactivity.

Physical activity is a health promoting factor, while inactivity or sedentary behavior is a risk factor for cardiovascular disease, type 2 diabetes, and mortality [50]. In the present questionnaire, the question regarding exercise did only ask about the hours per week for physical activity or exercise, but not if the activity was of moderate or high intensity. We could therefore not define a variable related to the World Health Organization 2020 guidelines on physical activity and sedentary behavior, of exercising for at least 150 minutes at moderate intensity or 75 minutes at high intensity every week [51]. We therefore focused on the risk factor and defined a variable of “inactivity”. Respondents who had done any amount of exercise in the past week were classified as “Inactivity = No”, and those who stated they had taken zero hours of exercise were classified as “Inactivity = Yes”.

### Statistical analysis

The data were analyzed using version 26 of SPSS for Mac, with the significance level set to 0.05. The results are shown as odds ratios (ORs) with 95% confidence intervals (CIs). Frequency tables including numbers of observations and percentages were created to assess the levels of stress, physical activity, and sleep disturbance in the study population. Descriptive statistics forage and BMI is presented as median, and minimum and maximum value. Logistic regression was used to investigate whether stress, physical inactivity, and sleep disturbance could be predictors for chronic pain. Adjustment for confounders was performed by first adding all potential confounders and then removing them one at a time with the highest p-value first. Confounders with p<0.250 were not removed, and those with 0.250≤p≤0.300 were removed only if this did not affect the OR by more than 10% [52]. All potential confounders presented in Table 2 were checked for in the process described above.

**Table 2:** Frequencies of confounders used in the analysis.

		Men		Women		Total	
		58.5%, n = 1029		41.5%, n = 730		n = 1759	
**Confounders**		%	n	%	n	%	n
**Education**	Computer science	61%	630	32%	236	49%	866
**Educational program at University level**							
	Medical	35%	359	59%	432	45%	791
	Nurse	1%	8	7%	52	3%	60
	Others	3%	32	1%	10	2%	42
**Using nicotine**	Yes (ref. No)	19%	191	7%	51	14%	242
**Asthma**	Yes (ref. No)	7%	73	6%	47	7%	120
**Diabetes**	Yes (ref. No)	1%	10	2%	11	1%	21
**PC use, nr of times >4h**	0	42%	431	74%	543	55%	974
**without a break**	1	17%	176	10%	73	14%	249
**in the previous week**	2–4	25%	260	9%	68	19%	328
	≥5	16%	162	6%	46	12%	208
**Watching TV/video,**	0	66%	675	75%	544	69%	1219
**nr of times >4h**	1	15%	152	13%	97	14%	249
**without a break**	2–4	14%	142	8%	59	11%	201
**in the previous week**	≥5	6%	60	4%	30	5%	90

Chronic pain at the one-year follow-up and at the four-year follow-up were used as dependent variables. One model was created with stress, non-restorative sleep, and physical inactivity as independent variables, and another with a variable including the interaction as independent variable. A variable with eight categories combining stress, sleep, and physical activity in all possible ways was constructed to check for interactions. In a statistical context, “interaction” refers to the possibility that the effect of a risk factor can vary depending on whether another risk factor is present. In our case we look at whether the OR presenting the effect of one risk factor, depends on the level of another factor.

The analyses were performed both with and without controlling for confounders.

## Results

At baseline, 1759 participants answered the questionnaire. There were more men (58.5%) than women in the study sample. The median age was 23 years (min: 19, max: 25) for both men and women. Median BMI was 22.8 (min: 15.2, max: 38.8) among men, 21.3 (min: 15.2, max: 40.4) among women, and 22.2 in the total group.

Frequencies of possible confounders are given in **Table 2**. Gender has been shown in previous studies to affect the probability of having chronic pain [53]. In a previous analysis of this database, the use of a computer for more than 4 hours without a break was shown to affect the probability of acute pain in the upper limbs [54]. Having watched TV/video for more than 4 hours without a break was also used as a confounder. Asthma and diabetes were the only comorbidities included in the questionnaire, and therefore were the only ones adjusted for. Body mass index (BMI) and nicotine use have been shown to affect pain levels [53]. University program was also used as a potential confounder.

Nearly half of the participants had experienced stress for more than 7 days in the past year. In the group, 14% reported inactivity, as they did not report any physical activity/exercise in the past week. Just over half had answered at least one of the sleep questions with “Several times a week” or “Every day”. Chronic pain in at least one of the locations was reported by 7% at the one-year follow-up and 8% at the four-year follow-up. More details of the baseline characteristics of the study population are given in **Table 3**.

**Table 3:** Characteristics of the study population.

		Men		Women		Total	
		58.5%, n = 1029		41.5%, n = 730		n = 1759	
		%	n	%	n	%	n
**Stress**	Yes (ref. No)	42%	436	59%	428	49%	864
**Physical activity, h/w**	Zero **(Inactivity)**	17%	175	10%	72	14%	247
	0.1–2.4	23%	227	26%	185	24%	412
	≥2.5	60%	602	64%	457	62%	1059
**Sleep**	Difficulty falling asleep	13%	138	11%	82	13%	220
	Frequent awakenings	2%	23	5%	38	4%	61
	Non-restorative sleep	37%	381	37%	273	37%	654
	Tired during the day	43%	447	46%	335	45%	782
**Pain, one-year follow-up**	Chronic pain upper back	2%	22	7%	44	4%	66
	Chronic pain lower back	3%	26	3%	18	3%	44
	Chronic pain arm	2%	17	4%	24	3%	41
	Chronic pain anywhere	6%	52	9%	60	7%	112
**Pain, four-year follow-up**	Chronic pain upper back	4%	31	5%	27	5%	58
	Chronic pain lower back	4%	25	3%	17	3%	42
	Chronic pain arm	4%	26	2%	12	2%	38
	Chronic pain anywhere	8.6%	62	8%	45	8%	107

### Results from regression analysis of one-year follow-up

Results from the regression model including main effects showed effect of all the three factors of interest (stress, non-restorative sleep and inactivity). Participants who reported stress for more than 7 consecutive days in the past 12 months had an adjusted OR of 1.6 (95% CI: 1.0–2.4) for developing chronic pain, those reporting non-restorative sleep a couple of times a week or every day had an adjusted OR of 1.5 (95% CI: 1.0–2.3), and those who said they had spent zero hours on exercise in the past week had an adjusted OR of 1.8 (95% CI: 1.1–3.0) **(Table 4, Model 1**).

**Table 4:** Results from logistic regression analysis of the outcome chronic pain at the one-year follow-up.

		Unadjusted			Adjusted^a^		
		OR	CI	p	OR	CI	p
**Model 1**							
**n = 1523, n = 1523**							
**Stress**	Yes (ref. No)	1.7	1.1–2.6	0.010	1.6	1.0–2.4	0.029
**Non-restorative sleep**	Yes (ref. No)	1.5	1.0–2.3	0.036	1.5	1.0–2.3	0.037
**Inactivity**	Yes (ref. No)	1.7	1.1–2.7	0.029	1.8	1.1–3.0	0.016
**Model 2**				<0.001			<0.001
**n = 1523, n = 1523**							
**Interaction variable**							
**Interactions (ref. no stress, no non-restorative sleep, no inactivity)**	Only stress	2.3	1.3–4.1	0.007	2.2	1.2–4.0	0.010
	Only non-rest. sleep	1.5	0.7–3.2	0.298	1.6	0.7–3.5	0.228
	Only inactivity	1.7	0.6–5.1	0.360	1.7	0.6–5.3	0.345
	Stress+inactivity	1.4	0.4–5.0	0.575	1.5	0.4–5.2	0.535
	Stress+non-rest. sleep	2.6	1.4–4.7	0.002	2.4	1.3–4.4	0.006
	Inact.+non-rest. sleep	6.2	2.3–17.0	0.001	6.9	2.5–19.2	<0.001
	All three	5.1	2.4–11.1	<0.001	5.2	2.4–11.5	<0.001
**Model 3**							
**n = 1523, n = 1523**							
**Stress**	Yes (ref. No)	1.7	1.1–2.5	0.016	1.6	1.0–2.4	0.042
**Inactivity**	Yes (ref. No)	1.7	1.0–2.7	0.039	1.8	1.1–2.9	0.024
Sleep disturbance^b^	Yes (ref. No)	0.9	0.5–1.6	0.671	0.8	0.5–1.5	0.518
Tiredness^c^	Yes (ref. No)	1.9	1.2–2.9	0.004	1.9	1.2–2.9	0.004

^a^ Included confounders in all models (Model 1-Model 3): gender, education

^b^ Yes = presence of at least one of “frequent awakenings” and “difficulty falling asleep”.

^c^ Yes = presence of at least one of “non-restorative sleep” and “tired during the daytime”.

OR = odds ratio, CI = confidence interval. The sample size in the unadjusted and adjusted model is given.

In model 2, Table 4, the model includes two-way interactions of the three factors to understand possible modifications. The reference group in the interaction model comprised those who had not been stressed, had reported restorative sleep, and had not been inactive. Strongest effect modifications were seen for those who had non-restorative sleep and were inactive, but had not been stressed (n = 30 at baseline), the adjusted OR for developing chronic pain at the one-year follow-up was 6.9. Those who reported all three conditions (stress, non-restorative sleep, and inactivity; n = 70 at baseline) showed an adjusted OR of 5.2, **Table 4**.

As a sensitivity analysis we also analyzed an alternative sleep variable model including sleep disturbance and tiredness. In the one-year follow-up stress and physical inactivity had similar ORs as in the model with non-restorative sleep. The variable “Sleep disturbance” did not show statistically significant results. “Tiredness” showed an adjusted OR of 1.9 for developing chronic pain, model 3 in **Table 4**.

The parametrization in the interaction model compares all combinations of stress, non-restorative sleep and inactivity with the group not being stressed, having restorative sleep and being active. To illustrate the models results for other group comparisons results are presented in **Table 5**. In the absence of stress, the OR for inactivity when non-restorative sleep was present was 4.3, and the OR for inactivity when non-restorative sleep was absent was 1.7. Again in the absence of stress, the OR for non-restorative sleep when inactivity was present was 4.1, and the OR for non-restorative sleep when inactivity was absent was 1.6, **Table 5**.

**Table 5:** Illustration of modification effect between the explanatory factors.

**Stress**		
Non-rest. sleep	Inactivity	OR stress^a^
-	-	2.2/1 = 2.2
	+	1.5/1.7 = 0.88
+	-	2.4/1.6 = 1.5
	+	5.2/6.9 = 0.75
**Non-restorative sleep**		
Stress	Inactivity	OR non-rest sleep^a^
**-**	-	1.6/1 = 1.6
	+	6.9/1.7 = 4.1
+	-	2.4/2.2 = 1.1
	+	5.2/1.5 = 3.5
**Inactivity**		
Stress	Non-rest. sleep	OR inactivity^a^
-	-	1.7/1 = 1.7
	+	6.9/1.6 = 4.3
+	-	1.5/2.2 = 0.68
	+	5.2/2.4 = 2.2

A minus sign represents absence of the factor and a plus sign represents presence of the factor. OR = odds ratio. ^a^ Calculated by dividing the odds for when the factor is present by the odds for where the factor is absent.

Logistic regression of the outcome chronic pain at the one-year follow-up.

### Results from regression analysis of four-year follow-up

Results at the four-year follow-up regression model including main effects only stress was a statistically significant predictor of chronic pain at this follow-up, **Table 6**. Exposure to stress at baseline showed an adjusted OR of 1.9 for developing chronic pain.

**Table 6:** Results from logistic regression analysis of the outcome chronic pain at the four-year follow-up.

		Unadjusted			Adjusted^a^		
		OR	CI	p	OR	CI	p
**Model 4**							
**n = 1251, n = 1245**							
**Stress**	Yes (ref. No)	1.8	1.2–2.6	0.008	1.9	1.3–2.9	0.002
**Non-restorative sleep**	Yes (ref. No)	1.0	0.7–1.6	0.858	1.1	0.7–1.6	0.813
**Inactivity**	Yes (ref. No)	1.3	0.8–2.2	0.340	1.2	0.7–2.0	0.615
**Model 5**				0.212			0.127
**n = 1251, n = 1245**							
**Interaction variable**							
**Interactions (ref. no stress, no non-restorative sleep, no inactivity)**	Only stress	1.9	1.1–3.3	0.026	2.1	1.2–3.7	0.011
	Only non-rest. sleep	1.2	0.6–2.5	0.668	1.2	0.6–2.6	0.581
	Only inactivity	1.3	0.4–3.9	0.658	0.9	0.3–3.3	0.928
	Stress+inactivity	2.0	0.7–5.4	0.194	2.1	0.8–6.0	0.146
	Stress+non-rest. sleep	1.7	0.9–3.1	0.080	1.9	1.0–3.4	0.041
	Inact.+non-rest. sleep	1.2	0.3–5.2	0.842	1.2	0.3–5.6	0.780
	All three	2.9	1.2–6.9	0.015	2.8	1.2–6.7	0.022
**Model 6**							
**n = 1251, n = 1245**							
**Stress**	Yes (ref. No)	1.7	1.1–2.6	0.013	1.9	1.2–2.9	0.005
**Inactivity**	Yes (ref. No)	1.3	0.8–2.2	0.373	1.1	0.7–1.9	0.668
**Sleep Disturbance^b^**	Yes (ref. No)	1.0	0.5–1.7	0.918	0.9	0.5–1.7	0.848
**Tiredness^c^**	Yes (ref. No)	1.2	0.8–1.9	0.356	1.3	0.8–1.9	0.305

^a^ Included confounders in all models (Model 4—Model 6): education, nicotine use

^b^ Yes = presence of at least one of “frequent awakenings” and “difficulty falling asleep”.

^c^ Yes = presence of at least one of “non-restorative sleep” and “tired during the daytime”.

OR = odds ratio, CI = confidence interval. The sample size in the unadjusted and adjusted model is given.

In model 5, the model includes two-way interactions of the three factors to understand possible modifications. The reference group in the interaction model comprised those who had not been stressed, had reported restorative sleep, and had not been inactive. At the four-year follow-up, the interaction variable was not statistically significant (p = 0.127), **Table 6**. Though, the OR = 2.1 of stress compared to reference group and all the factors present at the same time compared to reference group, were statistically significant, model 5, Table 6.

As a sensitivity analysis, we also analyzed an alternative sleep variable model including sleep disturbance and tiredness. In the four-year follow-up neither sleep disturbance nor tiredness showed statistically significant results (p = 0.848 and p = 0.305), **Table 6**.

### Sub-analysis of specific upper body pain locations

In the one-year follow-up when using chronic pain in the upper extremity as the dependent variable, stress was the only statistically significant predictor, with an adjusted OR of 2, **Table 7**.

**Table 7:** Results from logistic regression analysis of the outcome chronic pain at the one-year follow-up.

		Unadjusted			Adjusted^a^		
		OR	CI	p	OR	CI	p
**Model 7**	Upper back/neck						
**n = 1526, n = 1519**							
**Stress**	Yes (ref. No)	1.8	1.1–3.1	0.026	1.5	0.9–2.6	0.138
**Non-restorative sleep**	Yes (ref. No)	1.8	1.1–2.9	0.031	1.9	1.1–3.1	0.021
**Inactivity**	Yes (ref. No)	1.7	0.9–3.0	0.098	2.1	1.1–4.0	0.017
**Model 8**	Lower back						
**n = 1532, n = 1532**							
**Stress**	Yes (ref. No)	1.0	0.5–1.8	0.912	1.0	0.5–1.8	0.912
**Non-restorative sleep**	Yes (ref. No)	1.2	0.7–2.3	0.524	1.2	0.6–2.2	0.619
**Inactivity**	Yes (ref. No)	1.9	0.9–3.9	0.086	1.8	0.9–3.8	0.111
**Model 9**	Upper extremity						
**n = 1529, n = 1529**							
**Stress**	Yes (ref. No)	2.5	1.2–5.0	0.010	2.3	1.1–4.7	0.019
**Non-restorative sleep**	Yes (ref. No)	1.1	0.6–2.1	0.752	1.1	0.6–2.1	0.799
**Inactivity**	Yes (ref. No)	1.3	0.6–3.0	0.461	1.4	0.6–3.2	0.373

^a^ Included confounders in Model 7: gender, education, nicotine use, watching TV/video, Model 8: education, computer use, Model 9: gender, education

^b^ Yes = presence of at least one of “frequent awakenings” and “difficulty falling asleep”.

^c^ Yes = presence of at least one of “non-restorative sleep” and “tired during the daytime”.

OR = odds ratio, CI = confidence interval. The sample size in the unadjusted and adjusted model is given.

In the four-year follow-up when using chronic pain in the lower back as a dependent variable, stress was the only statistically significant predictor, with an adjusted OR of 2.9. Stress was also a statistically significant predictor for chronic pain in the upper extremity, with an adjusted OR of 2.5. Inactivity was a statistically significant predictor for pain in the upper extremity when unadjusted (OR 2.1), but not when adjusted. Results are given in, **Table 8.**

**Table 8:** Results from logistic regression analysis of the outcome chronic pain at the four-year follow-up.

		Unadjusted			Adjusted^a^		
		OR	CI	p	OR	CI	p
**Model 10**	Upper back/neck						
**n = 1251, n = 1251**							
**Stress**	Yes (ref. No)	1.2	0.7–2.1	0.512	1.2	0.7–2.0	0.512
**Non-restorative sleep**	Yes (ref. No)	1.0	0.6–1.8	0.912	1.0	0.6–1.8	0.912
**Inactivity**	Yes (ref. No)	1.4	0.7–2.8	0.312	1.4	0.7–2.8	0.312
**Model 11**	Lower back						
**n = 1251, n = 1251**							
**Stress**	Yes (ref. No)	2.5	1.3–4.9	0.007	2.9	1.5–5.7	0.002
**Non-restorative sleep**	Yes (ref. No)	0.8	0.4–1.5	0.442	0.7	0.4–1.4	0.326
**Inactivity**	Yes (ref. No)	1.0	0.4–2.4	0.952	0.8	0.3–2.1	0.708
**Model 12**	Upper extremity						
**n = 1245, n = 1245**							
**Stress**	Yes (ref. No)	2.3	1.1–4.6	0.022	2.5	1.2–5.2	0.012
**Non-restorative sleep**	Yes (ref. No)	1.0	0.5–1.9	0.997	1.0	0.5–1.9	0.930
**Inactivity**	Yes (ref. No)	2.1	1.0–4.5	0.047	1.9	0.9–4.2	0.089

^a^ Included confounders in Model 10: no confounders, Model 11: education, Model 12: education, nicotine use. ^b^ Yes = presence of at least one of “frequent awakenings” and “difficulty falling asleep”. ^c^ Yes = presence of at least one of “non-restorative sleep” and “tired during the daytime”.

OR = odds ratio, CI = confidence interval. The sample size in the unadjusted and adjusted model is given.

## Discussion

The results of this study showed that stress, non-restorative sleep, and inactivity were risk factors for chronic pain. Four years after baseline, only stress was a risk factor for chronic pain.

Reporting stress both at baseline and at the one-year follow-up increased the OR for developing chronic pain at the four-year follow-up, pointing to stress as a risk factor for chronic pain. This is in line with prior research [11] identifying stress as a short- and long-term risk factor[12, 55]. Several earlier studies [11–15] found stress to be a risk factor for neck and shoulder pain; our findings support a relation with pain in the upper extremity, but not specifically neck and shoulder pain.

Non-restorative sleep was a risk factor for chronic pain at the one-year follow-up, but at the four-year follow-up there were no statistically significant results, or even tendencies. These results imply that non-restorative sleep is a risk factor of chronic pain in the short term, but not in the long term. Earlier studies found that sleep disturbance predicts chronic pain in the short term [9], which is in line with our findings, but also in the long term [27, 29], which we could not show. There are a number of possible reasons for this discrepancy in results. Firstly, few other studies excluded individuals with any pain at baseline. Some excluded chronic pain, but not any pain [29], which may leave a risk of contamination by individuals who are close to developing chronic pain. Secondly, different results may have been due to different ways of measuring sleep disturbance. Our findings suggest that non-restorative sleep and daytime tiredness are predictors of chronic pain, while difficulty falling asleep and frequent awakenings are not. This may be because non-restorative sleep and daytime fatigue are effects of sleep disturbance, and the presence of effects may be a stronger predictor than sleep disturbance itself. Non-restorative sleep and daytime fatigue might stand for a more severe sleep disturbance. Another possible reason for not finding a relation between sleep disturbance and chronic pain could be that the stress question included difficulty falling asleep: “stress is defined as a condition where you feel tense, restless or anxious or can’t sleep at night because you keep thinking about problems”. However, if this was the true explanation then the OR for sleep disturbance would have been greater than 1 when stress was not included in the model and close to 1 when stress was included. In our study, the model without stress had an OR of 1.0 for sleep disturbance while the model which did include stress had an OR of 0.8.

Inactivity showed an OR of 1.8 for chronic pain at the one-year follow-up, but the results were not statistically significant at the four-year follow-up. Nevertheless, this suggests that inactivity may be a risk factor in the short term. Prior research is scarce on the subject, but the studies that do exist point in the same direction [36, 37]. Moreover, physical activity has been shown to have a moderate positive effect on sleep [56], which in turn might protect against the development of chronic pain.

Interaction effects were seen between non-restorative sleep and inactivity at the one-year follow-up in that they enhanced each other’s effects, making this an adverse combination. The negative effect of stress on chronic pain was strongest when none of the other factors were present; that is, in combination with good restorative sleep and activity.

Strengths of this study include the large number of participants and low drop-out rate. The longitudinal study design allowed us to make risk estimations for the development of chronic pain. The study sample consisted of only young people, who have not previously been thoroughly studied in this way and who have fewer comorbidities that may confound the results. Many previous studies focused on specific locations of chronic pain, while this study aimed to find more general results.

This study is based on a data from an earlier cohort study. Hence, its limitation is that we were only able to use already existing questions on pain, stress, and physical activity to fit our aim. This means that phrasing of questions, answer alternatives and periods over time that the questions covered, were already chosen before we planned our study.

One drawback of this is that the questionnaire did not ask about pain sites covering the whole body. We could only use the pain locations mentioned in the questionnaire (Pain/ache in the neck or upper part of the spine, Pain/ache in the lower part of the spine, Pain/ache in the shoulder/arm/wrist/hands) and the variable having pain in any of these sites. Hence, we did not have data on pain below the lower back. Therefore, at baseline in this study participants were pain free in all body parts above the lower extremities, while in lower extremities we had no knowledge if pain was present or not.

As mentioned above the definition of stress used in the questionnaire included “lying awake, thinking about problems”, which could imply a possible overlap with the sleep questions. Although we consider the overlap to be small, this should be remembered in the interpretation of our results.

The question on physical activity only took into account the last seven days, which may not have been representative of the participants’ usual level of exercise. It also did not specify intensity of activity, which made it hard to investigate whether level of exercise had an impact on pain development. It is possible that 75 minutes of high-intensity exercise could be equally favorable as 150 minutes at moderate intensity.

In the present study there was only information on asthma and diabetes diagnosis, but more comorbidity information and information on medication for the participants could have been valuable. Though, as this is a population study of a young cohort of university students the prevalence of co-morbidities and medications can be expected as low.

The data used in this study were collected over ten years ago, and it is possible that the prevalence of stress, sleep disturbance, and inactivity has changed since then. It is plausible that young adults today live slightly more stressed, sedentary lives [57]. However, there is no reason to believe that the effect of these factors on chronic pain would have changed.

## Conclusions

Stress, non-restorative sleep, and physical inactivity were risk factors for developing chronic pain one year after baseline, in a sample of young adults. Stress was also a risk factor four years after baseline. These findings suggest that non-restorative sleep and inactivity were risk factors in the short term and stress were a risk factor in both the short and the long term. A combination of non-restorative sleep and inactivity seems to additionally increase the odds for chronic pain.

This knowledge is important when working to decrease the incidence of chronic pain. As chronic pain is quite difficult to treat, prevention efforts are central. More research is required to fully understand how stress, sleep, and physical activity affect the development of chronic pain. For example, studies with a more diverse age group and different degrees of stress, sleep disturbance, and physical activity would be valuable.
